# Supplementary material for: Empirical methods for controlling false positives and estimating confidence in ChIP-Seq peaks
Source: BMC Bioinformatics. 2008 Dec 5;9:523. doi: 10.1186/1471-2105-9-523 (PMC2628906; doi:10.1186/1471-2105-9-523)
Supplement: Additional File 1 — A variety of html documents from the USeq web site detailing the available applications, their best usage, output file type descriptions, command line menus, etc. [file 1471-2105-9-523-S1.zip › USeqUserGuides/JavaDoc.zip!/JavaDoc/gata/menu/GATAFrame.html]

GATAFrame


|  |  |  |
| --- | --- | --- |
| **Overview** \| **Package** \| **Class** \| **Use** \| **Tree** \| **Deprecated** \| **Index** \| **Help** | |  |
| **PREV CLASS**   **NEXT CLASS** | **FRAMES**    **NO FRAMES**     **All Classes** |  |
| SUMMARY: NESTED \| FIELD \| CONSTR \| METHOD | DETAIL: FIELD \| CONSTR \| METHOD |  |


---


## gata.menu Class GATAFrame

```
java.lang.Object
  java.awt.Component
      java.awt.Container
          java.awt.Window
              java.awt.Frame
                  javax.swing.JFrame
                      gata.menu.GATAFrame
```

**All Implemented Interfaces:**: java.awt.image.ImageObserver, java.awt.MenuContainer, java.io.Serializable, javax.accessibility.Accessible, javax.swing.RootPaneContainer, javax.swing.WindowConstants

---

``` public class GATAFrame extends javax.swing.JFrame ```

**Author:**
:   Nix
    Frame holding all of the menues for running the GATAPlotter App. Is also a container for the
    aligner panel and any glyph/annotation panels. Sort of ugly!

**See Also:**: Serialized Form

---

**Field Summary**

**Fields inherited from class javax.swing.JFrame**

`EXIT_ON_CLOSE`

**Fields inherited from class java.awt.Frame**

`CROSSHAIR_CURSOR, DEFAULT_CURSOR, E_RESIZE_CURSOR, HAND_CURSOR, ICONIFIED, MAXIMIZED_BOTH, MAXIMIZED_HORIZ, MAXIMIZED_VERT, MOVE_CURSOR, N_RESIZE_CURSOR, NE_RESIZE_CURSOR, NORMAL, NW_RESIZE_CURSOR, S_RESIZE_CURSOR, SE_RESIZE_CURSOR, SW_RESIZE_CURSOR, TEXT_CURSOR, W_RESIZE_CURSOR, WAIT_CURSOR`

**Fields inherited from class java.awt.Component**

`BOTTOM_ALIGNMENT, CENTER_ALIGNMENT, LEFT_ALIGNMENT, RIGHT_ALIGNMENT, TOP_ALIGNMENT`

**Fields inherited from interface javax.swing.WindowConstants**

`DISPOSE_ON_CLOSE, DO_NOTHING_ON_CLOSE, HIDE_ON_CLOSE`

**Fields inherited from interface java.awt.image.ImageObserver**

`ABORT, ALLBITS, ERROR, FRAMEBITS, HEIGHT, PROPERTIES, SOMEBITS, WIDTH`


**Constructor Summary**

`GATAFrame(GATAParams gataParams)`


| **Method Summary** | |
| --- | --- |
| `javax.swing.JMenuItem` | `addScaleRuler(javax.swing.JMenu menu, java.lang.String label)` |
| `javax.swing.JMenuItem` | `addScaleTrackColors(javax.swing.JMenu menu)` |
| `javax.swing.JMenuItem` | `addShowHide(javax.swing.JMenu menu, java.lang.String label, boolean color)` |
| `javax.swing.JMenuItem` | `addShowHideLabel(javax.swing.JMenu menu, java.lang.String label)` |
| `javax.swing.JMenuItem` | `addShowHideWithPath(javax.swing.JMenu menu, java.lang.String label, java.lang.String path)` |
| `javax.swing.JMenuItem` | `addTrack(javax.swing.JMenu menu, java.lang.String label, boolean color)` |
| `void` | `colorizeGenericGlyphs(int colorizer)`             helper method, 0 = linear, 1 = log10, 2 = lnx, 3= reset to solid 4= log2 |
| `void` | `completeAnnotationMenu(boolean gffRefPres, boolean gffCompPres, AnnoSpecParams refParams, AnnoSpecParams compParams)`             must be called after the initial creation when the user has possibly loaded generic annotation |
| `void` | `reallyRedrawAnnotationPanel(AnnoSpecParams asp)` |
| `void` | `redrawAlignPanel()` |
| `void` | `redrawAnnotationPanels()`             basically draws annotation twice, once to estimate the size, the second time for real |
| `void` | `setAlignPanel(AlignPanel panel)` |
| `void` | `setCompAnnotation(Annotation a)`             needed to add later since annotation doesn't exist when ToolFrame is created |
| `void` | `setCompGlyphPanel(GlyphPanel panel)` |
| `void` | `setRefAnnotation(Annotation a)`             needed to add later since annotation doesn't exist when ToolFrame is created |
| `void` | `setRefGlyphPanel(GlyphPanel panel)` |
| `void` | `setWindowsVisibility(boolean setting)`             Add new windows/panels here to enable them to be shown or hidden in sync with the main frame |

**Methods inherited from class javax.swing.JFrame**

`getAccessibleContext, getContentPane, getDefaultCloseOperation, getGlassPane, getJMenuBar, getLayeredPane, getRootPane, isDefaultLookAndFeelDecorated, remove, setContentPane, setDefaultCloseOperation, setDefaultLookAndFeelDecorated, setGlassPane, setIconImage, setJMenuBar, setLayeredPane, setLayout, update`

**Methods inherited from class java.awt.Frame**

`addNotify, getCursorType, getExtendedState, getFrames, getIconImage, getMaximizedBounds, getMenuBar, getState, getTitle, isResizable, isUndecorated, remove, removeNotify, setCursor, setExtendedState, setMaximizedBounds, setMenuBar, setResizable, setState, setTitle, setUndecorated`

**Methods inherited from class java.awt.Window**

`addPropertyChangeListener, addPropertyChangeListener, addWindowFocusListener, addWindowListener, addWindowStateListener, applyResourceBundle, applyResourceBundle, createBufferStrategy, createBufferStrategy, dispose, getBufferStrategy, getFocusableWindowState, getFocusCycleRootAncestor, getFocusOwner, getFocusTraversalKeys, getGraphicsConfiguration, getInputContext, getListeners, getLocale, getMostRecentFocusOwner, getOwnedWindows, getOwner, getToolkit, getWarningString, getWindowFocusListeners, getWindowListeners, getWindowStateListeners, hide, isActive, isAlwaysOnTop, isFocusableWindow, isFocusCycleRoot, isFocused, isLocationByPlatform, isShowing, pack, postEvent, removeWindowFocusListener, removeWindowListener, removeWindowStateListener, setAlwaysOnTop, setBounds, setCursor, setFocusableWindowState, setFocusCycleRoot, setLocationByPlatform, setLocationRelativeTo, show, toBack, toFront`

**Methods inherited from class java.awt.Container**

`add, add, add, add, add, addContainerListener, applyComponentOrientation, areFocusTraversalKeysSet, countComponents, deliverEvent, doLayout, findComponentAt, findComponentAt, getAlignmentX, getAlignmentY, getComponent, getComponentAt, getComponentAt, getComponentCount, getComponents, getComponentZOrder, getContainerListeners, getFocusTraversalPolicy, getInsets, getLayout, getMaximumSize, getMinimumSize, getMousePosition, getPreferredSize, insets, invalidate, isAncestorOf, isFocusCycleRoot, isFocusTraversalPolicyProvider, isFocusTraversalPolicySet, layout, list, list, locate, minimumSize, paint, paintComponents, preferredSize, print, printComponents, remove, removeAll, removeContainerListener, setComponentZOrder, setFocusTraversalKeys, setFocusTraversalPolicy, setFocusTraversalPolicyProvider, setFont, transferFocusBackward, transferFocusDownCycle, validate`

**Methods inherited from class java.awt.Component**

`action, add, addComponentListener, addFocusListener, addHierarchyBoundsListener, addHierarchyListener, addInputMethodListener, addKeyListener, addMouseListener, addMouseMotionListener, addMouseWheelListener, bounds, checkImage, checkImage, contains, contains, createImage, createImage, createVolatileImage, createVolatileImage, disable, dispatchEvent, enable, enable, enableInputMethods, firePropertyChange, firePropertyChange, firePropertyChange, firePropertyChange, firePropertyChange, firePropertyChange, getBackground, getBounds, getBounds, getColorModel, getComponentListeners, getComponentOrientation, getCursor, getDropTarget, getFocusListeners, getFocusTraversalKeysEnabled, getFont, getFontMetrics, getForeground, getGraphics, getHeight, getHierarchyBoundsListeners, getHierarchyListeners, getIgnoreRepaint, getInputMethodListeners, getInputMethodRequests, getKeyListeners, getLocation, getLocation, getLocationOnScreen, getMouseListeners, getMouseMotionListeners, getMousePosition, getMouseWheelListeners, getName, getParent, getPeer, getPropertyChangeListeners, getPropertyChangeListeners, getSize, getSize, getTreeLock, getWidth, getX, getY, gotFocus, handleEvent, hasFocus, imageUpdate, inside, isBackgroundSet, isCursorSet, isDisplayable, isDoubleBuffered, isEnabled, isFocusable, isFocusOwner, isFocusTraversable, isFontSet, isForegroundSet, isLightweight, isMaximumSizeSet, isMinimumSizeSet, isOpaque, isPreferredSizeSet, isValid, isVisible, keyDown, keyUp, list, list, list, location, lostFocus, mouseDown, mouseDrag, mouseEnter, mouseExit, mouseMove, mouseUp, move, nextFocus, paintAll, prepareImage, prepareImage, printAll, removeComponentListener, removeFocusListener, removeHierarchyBoundsListener, removeHierarchyListener, removeInputMethodListener, removeKeyListener, removeMouseListener, removeMouseMotionListener, removeMouseWheelListener, removePropertyChangeListener, removePropertyChangeListener, repaint, repaint, repaint, repaint, requestFocus, requestFocusInWindow, reshape, resize, resize, setBackground, setBounds, setComponentOrientation, setDropTarget, setEnabled, setFocusable, setFocusTraversalKeysEnabled, setForeground, setIgnoreRepaint, setLocale, setLocation, setLocation, setMaximumSize, setMinimumSize, setName, setPreferredSize, setSize, setSize, setVisible, show, size, toString, transferFocus, transferFocusUpCycle`

**Methods inherited from class java.lang.Object**

`equals, getClass, hashCode, notify, notifyAll, wait, wait, wait`

**Methods inherited from interface java.awt.MenuContainer**

`getFont, postEvent`

**Constructor Detail**

### GATAFrame

```
public GATAFrame(GATAParams gataParams)
```


**Method Detail**

### setRefAnnotation

```
public void setRefAnnotation(Annotation a)
```

:   needed to add later since annotation doesn't exist when ToolFrame is created

---


### setCompAnnotation

```
public void setCompAnnotation(Annotation a)
```

:   needed to add later since annotation doesn't exist when ToolFrame is created

---


### completeAnnotationMenu

```
public void completeAnnotationMenu(boolean gffRefPres,
                                   boolean gffCompPres,
                                   AnnoSpecParams refParams,
                                   AnnoSpecParams compParams)
```

:   must be called after the initial creation when the user has possibly loaded generic annotation

---


### addShowHide

```
public javax.swing.JMenuItem addShowHide(javax.swing.JMenu menu,
                                         java.lang.String label,
                                         boolean color)
```

---


### addScaleRuler

```
public javax.swing.JMenuItem addScaleRuler(javax.swing.JMenu menu,
                                           java.lang.String label)
```

---


### addScaleTrackColors

```
public javax.swing.JMenuItem addScaleTrackColors(javax.swing.JMenu menu)
```

---


### addShowHideWithPath

```
public javax.swing.JMenuItem addShowHideWithPath(javax.swing.JMenu menu,
                                                 java.lang.String label,
                                                 java.lang.String path)
```

---


### addShowHideLabel

```
public javax.swing.JMenuItem addShowHideLabel(javax.swing.JMenu menu,
                                              java.lang.String label)
```

---


### addTrack

```
public javax.swing.JMenuItem addTrack(javax.swing.JMenu menu,
                                      java.lang.String label,
                                      boolean color)
```

---


### colorizeGenericGlyphs

```
public void colorizeGenericGlyphs(int colorizer)
```

:   helper method, 0 = linear, 1 = log10, 2 = lnx, 3= reset to solid 4= log2

---


### redrawAlignPanel

```
public void redrawAlignPanel()
```

---


### redrawAnnotationPanels

```
public void redrawAnnotationPanels()
```

:   basically draws annotation twice, once to estimate the size, the second time for real

---


### reallyRedrawAnnotationPanel

```
public void reallyRedrawAnnotationPanel(AnnoSpecParams asp)
```

---


### setAlignPanel

```
public void setAlignPanel(AlignPanel panel)
```

---


### setCompGlyphPanel

```
public void setCompGlyphPanel(GlyphPanel panel)
```

---


### setRefGlyphPanel

```
public void setRefGlyphPanel(GlyphPanel panel)
```

---


### setWindowsVisibility

```
public void setWindowsVisibility(boolean setting)
```

:   Add new windows/panels here to enable them to be shown or hidden in sync with the main frame


---


|  |  |  |
| --- | --- | --- |
| **Overview** \| **Package** \| **Class** \| **Use** \| **Tree** \| **Deprecated** \| **Index** \| **Help** | |  |
| **PREV CLASS**   **NEXT CLASS** | **FRAMES**    **NO FRAMES**     **All Classes** |  |
| SUMMARY: NESTED \| FIELD \| CONSTR \| METHOD | DETAIL: FIELD \| CONSTR \| METHOD |  |


---
